# Prognostic Nutritional Index and CRP, age, platelet count, albumin level score in predicting mortality and intensive care unit admission for COVID-19

**DOI:** 10.2217/bmm-2021-0337

**Published:** 2021-11-17

**Authors:** Mehmet Bayram, Ozgur Yildirim, Raye S Ozmen, Beyza Soylu, Ahmet S Dundar, Ali R Koksal, Iskender Ekinci, Murat Akarsu, Omur Tabak

**Affiliations:** ^1^Department of Gastroenterology, Health Sciences University Kanuni Sultan Süleyman Training & Research Hospital, İstanbul, 34303, Turkey; ^2^Department of Internal Medicine, Health Sciences University Kanuni Sultan Süleyman Training & Research Hospital, İstanbul, 34303, Turkey; ^3^Department of Gastroenterology & Hepatology, Tulane University School of Medicine, New Orleans, LA, 70112, USA

**Keywords:** CAPA score, COVID-19, ICU admission, mortality, prognostic nutritional index

## Abstract

**Aim:** In our study, we investigated the efficiency of the prognostic nutritional index (PNI) score and the CRP, age, platelet count, albumin level (CAPA) score predicting mortality and intensive care unit (ICU) admission in COVID-19 disease. **Materials & methods:** PNI and CAPA score of patients confirmed with COVID-19 calculated by using the complete blood count and biochemical parameters at admission to the hospital, in predicting the COVID-19-associated mortality and ICU admission were analyzed. **Results:** PNI and CAPA scores in predicting mortality were detected as AUC: 0.67 (p < 0.001), AUC: 0.71 (p < 0.001), respectively. For predicting ICU admission AUC was 0.66 (p < 0.001), AUC was 0.77 (p < 0.001), respectively. **Conclusion:** PNI and CAPA scores are effective scores in COVID-19, with CAPA score being better in predicting mortality and ICU admission.

## Lay abstract

The COVID-19 pandemic is a global health problem that affects all societies. In order to deal with this urgent situation, the rapid spread of the disease in outbreaks requires categorizing patients according to risk group and regulating follow-up and use of resources accordingly. Effective, practical and inexpensive biomarkers are needed. We present to you the CAPA score calculated from CRP, age, platelet count, albumin levels, which is an effective score in predicting mortality and ICU admission in COVID-19.

The SARS-Cov-2 virus was detected for the first time in China in December 2019 and subsequently spread to more than 100 million people in more than 200 countries, causing a global pandemic. After the pandemic was declared by WHO in March 2020, the name of this disease was determined as COVID-19. COVID-19 is a disease that can progress in a wide spectrum from asymptomatic mild illness to severe lung disease [1,2]. Inflammation plays an important role in the pathogenesis of viral pneumonia [3]. Dysregulated or uncontrolled inflammation in the immune response is also crucial in the pathogenesis of the disease in COVID-19. Prognostic Nutritional Index (PNI) is a marker of inflammatory and nutritional status that can be calculated by lymphocyte and albumin levels and has been associated with poor prognosis in cancer, cardiovascular disease, pulmonary thromboembolism and stroke [4–7]. PNI was found to be associated with mortality in COVID-19 disease in multivariate analysis studies [8]. Age is one of the major risk factors for severe COVID-19 disease [9,10]. CRP (C-reactive protein) is one of the most basic biomarkers indicating mortality in patients with sepsis and intensive care unit (ICU) [11]. CRP is associated with poor prognosis and in hospital death in COVID-19 disease [12,13]. Thrombocytopenia occurs at a rate of 5–41% during the course of COVID-19, and typically mild thrombocytopenia is 100–150 × 10^9^/l [14]. Thrombocytopenia is associated with the severity of disease and mortality for the COVID-19 [15,16]. COVID-19 is a disease that critically affects elderly patients and people with previous comorbidities. COVID-19 pandemic has shown that patient follow-up and use of resources should be organized by categorizing patients according to risk groups. Therefore, to predict the course of the disease, useful, reliable and simple scoring systems that can be used for each patient are needed. In our study, we investigated the effectiveness of the CAPA (an abbreviation formed by the first letters of the words CRP, age, platelet and albumin) score, which we created using the patient's CRP level, age, platelet count and albumin level, in predicting intensive care need and mortality in COVID-19 disease, in addition to the PNI score.

## Materials & methods

**Figure 1.**
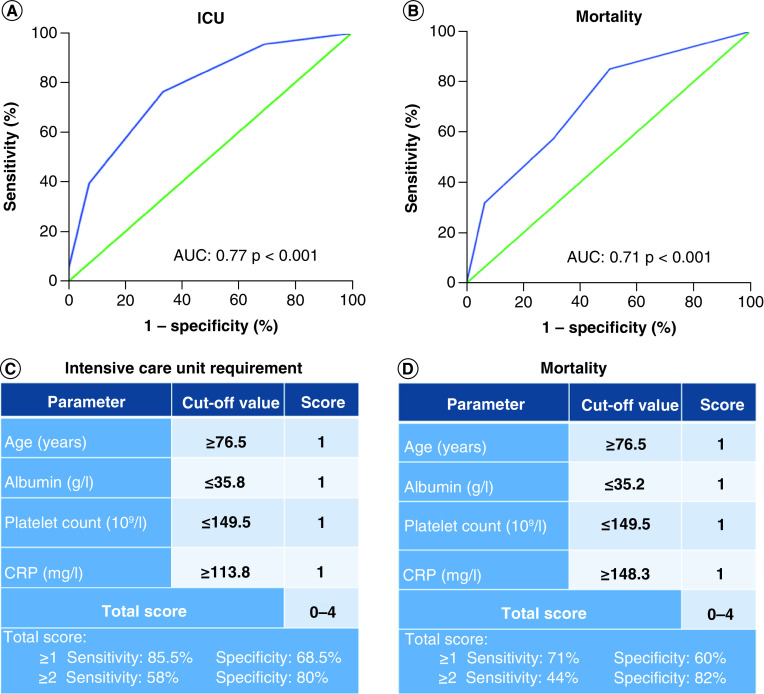
Receiver operating characteristic curve analysis of CRP, age, platelet count and albumin score for prediction of mortality and intensive care unit admission. **(A)** ROC analysis of CAPA score for intensive care unit admission. **(B)** ROC analysis of CAPA score for mortality. **(C)** Calculation of CAPA score for predicting intensive care unit admission. **(D)** Calculation of CAPA score for predicting mortality. AUC: Area under the ROC curve; CRP: C-reactive protein.

The study was conducted as a retrospective cross-sectional research using medical records of inpatients with a positive COVID-19 real-time PCR (polymerase chain reaction) test treated at Kanuni Sultan Suleyman Training and Research Hospital (a tertiary care hospital in Istanbul, Turkey) between 1 September 2020 and 31 December 2020. Ethical approval was obtained from the local ethics committee for the study (no: KAEK/ 2021.03.70) and the study was conducted in accordance with the Helsinki declaration. A total of 748 patients aged 18 years and older, who were hospitalized and followed up, with Wuhan variant positive COVID-19 PCR test, were included in the study. Outpatients and patients under the age of 18 were excluded from the study. Complete blood count results and biochemical parameters of the patients at their admission to the hospital were analyzed. The PNI was calculated as serum albumin (g/l) + 5× lymphocyte count (10^9^/l). In our study, we planned to make a simple qualitative scoring system for values above the cutoff by determining the independent factors affecting mortality and ICU admission with logistic regression analysis. While creating the CAPA score, individual optimal cutoffs were determined for CRP, age, platelet count and albumin, which appear as independent predictor values for ICU admission and mortality in the multivariate analysis model. A total scoring system was created by giving 1 point for the values above or below of these cutoffs (Figure 1). ICU admissions and mortality due to the disease were recorded. Together with other hematological and biochemical parameters, the efficiency of PNI and CAPA scores in predicting mortality and ICU admission was evaluated. Complete blood count parameters were determined by a hematology analyzer (Mindray BC-6800 Plus). Biochemical parameters were measured by the Roche Cobas Integra 800 (Roche Diagnostic Limited, Switzerland) device.

### The universe & power

The universe of the study consists a total of 748 patients aged 18 years and older, who were hospitalized and followed up, with Wuhan variant positive COVID-19 PCR test. Since it is a retrospective study and a scoring system that has not been evaluated before, *post hoc* power analysis was performed in the study. G*Power version 3.1.9.2 was used for analysis. When effect size was accepted as >0.80 and alpha error: <0.01, the power of the study was found to be >95%.

### Statistical study

All analyses were performed using SPSS version 21.0 for Windows (IBM Inc, IL, USA) and using GraphPad Prism version 8.0.0 for Windows (GraphPad Software, CA, USA). The variables were investigated using visual (histograms) and analytical methods (Kolmogorov–Smirnov/Shapiro–Wilk tests) to determine whether or not they were normally distributed. The Chi-square test was used for comparisons of categorical variables. Continuous variables were shown as mean ± standard deviation or median (interquartile range) according to their distribution pattern. Ordinal variables and continuous variables that do not have normal distribution were compared by the Mann–Whitney *U* test. The Student's *t*-test was used to evaluate differences between the two groups in normally distributed continuous variables. For multivariate analysis, the possible factors identified by univariate analysis or recent studies were further entered into the logistic regression analysis to determine independent predictors of undergoing to ICU and death. Variables that did not make a significant contribution were excluded from the model using the backward likelihood ratio stepwise method. Hosmer–Lemeshow goodness-of-fit statistics were used to assess the model. To find optimal cutoffs and build a clinical score, individual receiver operating characteristic curve (ROC) analysis was performed for all independent risk factors. Sensitivity and specificity performances were determined for the CAPA score as well as other risk factors. A value of p < 0.05 (two-sided) was considered statistically significant.

## Results

**Figure 2.**
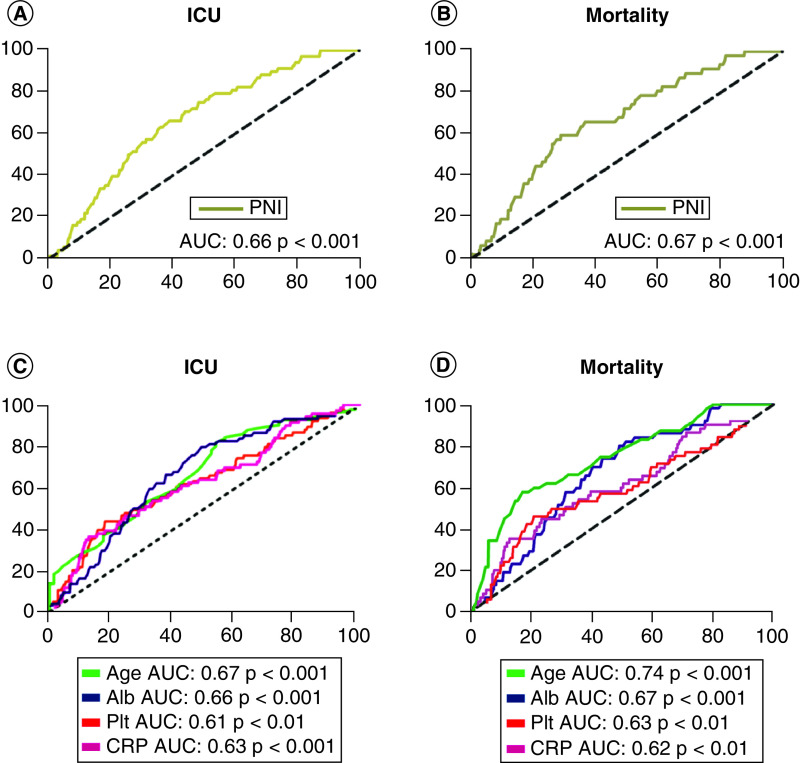
Receiver operating characteristic curve analysis of age, albumin level, platelet count, C-reactive protein and prognostic nutritional index for prediction of mortality and ICU admission. **(A)** ROC analysis of PNI for ICU admission. **(B)** ROC analysis of PNI for mortality. **(C)** ROC analysis of the parameters that constitute the CRP, age, platelet count, albumin level score for ICU admission. **(D)** ROC analysis of the parameters that constitute the CRP, age, platelet count, albumin level score for mortality. AUC: Area under the ROC curve; CRP: C-reactive protein; ICU: Intensive care unit.

In our study, the data of 748 patients diagnosed with COVID-19, including 410 men and 338 women, were investigated. In the data of our study, the number of patients referred to ICU was 68 (9.1%). The disease resulted in mortality in 47 (6.2%) of 748 patients. The patients included in the study were grouped in terms of mortality as survival/death. Also, patients were evaluated according to their referral to intensive care by grouping them as ICU (+)/ICU (-). There was a statistically significant difference in age (p: 0.001), platelet count (p: 0.005), CRP (p: 0.001), creatinine (p: 0.001), AST (p: 0.021), albumin (p: 0.001) and PNI (p: 0.001) values between ICU (+) and ICU (-) groups. Likewise, a statistically significant difference was found between the survival and death groups determined according to mortality in terms of age (p: 0.001), platelet count (p: 0.009), CRP (p: 0.031), creatinine (p: 0.001), AST (p: 0.014), albumin (p: 0.001) and PNI (p: 0.001) values. Clinical and biochemical parameters in all groups are shown in Table 1. In the univariate analysis, age, CRP, platelet count, PNI and albumin were found to be significant in showing the risk of mortality and ICU admission. In regression analysis, age, platelet count and CRP level were found to be independent risk factors for mortality and ICU admission (Tables 2 & 3). The results of ROC analysis performed for these parameters (age, CRP, platelet count, albumin and PNI score) in predicting mortality and ICU admission are given in Tables 4 & 5 & Figure 2. AUC was 0.67 (p < 0.001) with 59.6% specificity and 71.2% sensitivity for PNI ≤38.5 in predicting mortality with PNI score. AUC was 0.66 (p < 0.001) with 63% specificity and 64% sensitivity for PNI ≤39.9 in predicting the ICU admission with PNI score (Figure 2). AUC was 0.71 (p < 0.001) in predicting mortality with the CAPA score, 71% specificity and 60% sensitivity for CAPA ≥1, and 44% specificity and 82% sensitivity for CAPA ≥2. In predicting ICU admission, AUC was 0.77 (p < 0.001) with 85.5% specificity and 68.5% sensitivity for CAPA ≥1, and 58% specificity and 80% sensitivity for CAPA ≥2 (Figure 1).

**Table 1.** Main clinical and biochemical characteristics of patients grouped according to mortality and ICU admission.

Characteristics	Mortality			ICU		
	Survived (n: 701)	Death (n: 47)	p-value	ICU (–) (n: 680)	ICU (+) (n: 68)	p-value
Sex (male/female)	319/382	19/28	0.54	312/368	26/42	0.22
Age (year)	61 ± 15.1	74 ± 12.7	**0.001**	61 ± 15.2	71 ± 13.1	**0.001**
WBC (10^3^/μl)	7.4 ± 3.4	7.6 ± 3.9	0.71	7.4 ± 3.4	7.6 ± 3.4	0.59
Neutrophil (10^3^/μl)	4.7 (3.3–7.0)	5.2 (3.2–7.4)	0.48	4.6 (3.3–7)	5.4 (3.5–7.3)	0.17
Lymphocyte (10^3^/μl)	1.3 ± 0.6	1.1 ± 0.7	0.09	1.3 ± 0.65	1.1 ± 0.66	**0.08**
Hgb (mg/dl)	12.7 ± 1.9	12.3 ± 1.8	0.15	12.7 ± 1.9	12.4 ± 2	0.14
Platelet (10^3^/μl)	221 ± 90	185 ± 91	**0.009**	222 ± 91	189 ± 83	**0.005**
CRP (mg/dl)	62 (24–111)	97 (33–160)	**0.003**	62 (24–110)	99 (34–162)	**0.001**
Cre (mg/dl)	0.9 (0.7–1.1)	1.2 (0.8–1.7)	**0.001**	0.9 (0.7–1.1)	1.2 (0.8–1.7)	**0.001**
AST (U/l)	28 (21–38)	35 (24–42)	**0.014**	28 (21–38)	34 (24–46)	**0.021**
ALT (U/l)	21 (14–31)	18 (13–25)	0.16	21 (14–31)	19 (15–34)	0.96
Alb (mg/dl)	35 ± 4.6	32 ± 3.2	**0.001**	35 ± 4.7	33 ± 3.4	**0.001**
PNI	42 ± 6.2	38 ± 5	**0.001**	42 ± 6.2	38 ± 4.8	**0.001**

Values are presented using means ± standard deviations for normally distributed and medians and first and third quartiles in the brackets for the non-normally distributed variables.

Chi-Square test was used to compare gender ratios, Student's *t*-test was used to compare averages suitable for normal distribution, and Mann–Whitney U test was used to compare non-distributed variables; WBC: White blood cell.

Alb: Albumin; ALT: Alanine aminotransferase; AST: Aspartate aminotransferase; Cre: Creatinin; CRP: C-reactive protein; Hgb: Hemoglobin; ICU: Intensive care unit; Plt: Platelet count, PNI: Prognostic Nutritional Index.

**Table 2.** Univariate and multivariate analysis table for ICU admission.

Characteristics	Univariate analysis				Multivariate analysis			
		95% CI				95% CI		
	OR	Lower bound	Upper bound	p-value	OR	Lower bound	Upper bound	p-value
Age (years)	1.048	1.029	1.069	**0.001**	1.043	1.023	1.064	**0.001**
Albumin	0.895	0.847	0.945	**0.001**	0.940	0.884	1.001	0.052
Platelet	0.995	0.992	0.998	**0.005**	0.995	0.991	0.998	**0.004**
CRP	1.006	1.003	1.009	**0.001**	1.006	1.003	1.010	**0.001**
Sex	1.370	0.821	2.285	0.228				
PNI	0.914	0.876	0.954	**0.001**				
AST	1.001	0.994	1.008	0.782				
Cre	1.059	0.930	1.204	0.387				
Lymp	0.606	0.387	0.949	**0.029**				

AST: Aspartate aminotransferase; Cre. Creatinine; CRP: C-reactive protein; ICU: Intensive care unit; Lymp: Lymphocyte; PNI: Prognostic Nutritional Index.

**Table 3.** Univariate and multivariate analysis table for mortality.

Characteristics	Univariate analysis				Multivariate analysis			
		95% CI				95% CI		
	OR	Lower bound	Upper bound	p-value	OR	Lower bound	Upper bound	p-value
Age (years)	1.070	1.044	1.097	**0.001**	1.064	1.037	1.092	**0.001**
Albumin	0.886	0.831	0.944	**0.001**	0.936	0.869	1.009	0.086
Platelet	0.994	0.990	0.999	**0.008**	0.994	0.990	0.998	**0.008**
CRP	1.006	1.002	1.009	**0.001**	1.006	1.002	1.010	**0.004**
Sex	1.231	0.675	2.225	0.499				
PNI	0.911	0.866	0.957	**0.001**				
AST	1.001	0.994	1.009	0.695				
Cre	1.084	0.943	1.247	0.255				

AST: Aspartate aminotransferase; Cre: Creatinine; CRP: C-reactive protein; PNI: Prognostic Nutritional Index.

**Table 4.** Predictors for mortality in hospitalized patients with COVID-19 infection.

Characteristics	cutoff	Sensitivity	Specificity	AUC	p-value
Age (years)	≥76.5	57.4	83.2	74	<0.001
Albumin	≤35.25	78.7	53.1	67.6	<0.001
Platelet	≤149.5	49	82	63.1	<0.001
CRP	≥148.3	36	88	62.7	<0.001
PNI	≤38.75	59.6	71.2	66.9	<0.001

CRP: C-reactive protein; PNI: Prognostic Nutritional Index.

**Table 5.** Predictors for ICU admission in hospitalized patients with COVID-19 infection.

Characteristics	Cutoff	Sensitivity	Specificity	AUC	p-value
Age (years)	≥76.5	45	83	67.6	<0.001
Albumin	≤35.85	83	47	66.6	<0.001
Platelet	≤149.5	42.6	82.2	61.6	<0.001
CRP	≥113.8	45.6	77	63	<0.001
PNI	≤39.95	63.2	64	66.5	<0.001

CRP: C-reactive protein; ICU: Intensive care unit; PNI: Prognostic Nutritional Index.

## Discussion

PNI, which we evaluated in the course of COVID-19, was first identified as a predictor of postoperative complications after gastrointestinal surgery and was brought into clinical use [17]. Low serum albumin level, which is an important mortality indicator for patients followed up in intensive care units, was also found to be associated with poor prognosis in pneumonia caused by COVID-19 [18,19]. In the course of inflammation caused by COVID-19, a severe clinical condition known as cytokine storm and caused by systemic hyperinflammation may occur. Production of IL-6, IL-8, TNF-α, which are virus-related inflammatory factors, is made from the endothelium and lymphocyte [20]. IL-6 and TNF-α inhibit albumin synthesis [21]. Large amounts of these cytokines are released in a cytokine storm [22]. In addition, extravascular escape and increased pulmonary edema are associated with hypoalbuminemia. In the studies conducted, the serum albumin level and acute respiratory distress syndrome (ARDS) development in patients show an inverse proportion [23]. Hypoalbuminemia may show hypermetabolism and heavy consumption in critically ill patients [24].

Immunity to viral infections is mainly based on lymphocytes [20]. Lymphopenia is associated with the overactivation of the inflammatory cascade [25]. Lymphocyte apoptosis develops secondary to increased TNF-α and IL-6 levels in a cytokine storm, and COVID-19 causes down-regulation in genes that cause T lymphocyte proliferation [26,27]. In the literature, the PNI score has been associated with mortality in COVID-19 [8]. In our study also, the PNI score was determined as an effective marker in predicting mortality and ICU admission in COVID-19 disease.

In COVID-19-associated infection, thrombocyte production and destruction increase together, but a mild thrombocytopenia is usually detected in patients [28]. This thrombocytopenia can be explained by the elimination of thrombocytes from the reticuloendothelial system, the development of platelet autoantibody formations due to endothelial damage, splenic secretion, and megakaryocyte suppression in the bone marrow [28].

In COVID-19, hospitalization, ICU admission and mortality rates are higher in patients over 65 years of age [29]. The immune response is affected with advanced age, and the frequency of infections increases in elderly patients [30]. Aging-related changes may occur in the maturation and function of B and T lymphocytes [31]. C-reactive protein, known as the short pentraxin protein, is one of the most basic inflammatory biomarkers in clinical use. CRP acts as part of innate immunity, which binds to the pathogen and helps eliminate the pathogen by phagocytic cells [32]. CRP is an independent risk factor for severe COVID-19 disease. When serum CRP levels increase by 1U, the risk of severe disease increases by 0.06% [33].

In the course of COVID-19 disease, age, CRP level and albumin level correlate with high consistency with the severity of the disease [34]. In the light of this information, the CAPA score calculated using CRP, age, platelet count and albumin levels, which seem to be an independent predictor for ICU admission and mortality in our multivariate analysis model, is effective in predicting mortality and the ICU admission, and is better than all other parameters, especially in predicting the ICU admission. This study evaluating the effectiveness of the CAPA score in COVID-19 disease is the first in the literature. Patients with high CAPA scores should be treated with a more energetic and multidisciplinary approach and evaluated for further treatment options. There is a need for verification of the CAPA score with studies in different centers with larger patient samples.

Limitations: the limitations of our study were that it was a single-center study, it was conducted retrospectively, there was no healthy control group in the study, and the sample size was low compared with the pandemic. The use of logistic regression analysis in calculating the CAPA score limits the practical use of scoring, and further studies on the value of the score are needed.

## Conclusion

Risk grouping with simple, inexpensive and practical scoring systems facilitates treatment and follow-up approaches. With this study, we present to you the CAPA score calculated from CRP, age, platelet count, albumin levels as an effective score in predicting mortality and ICU admission in COVID-19.

**Summary points**

- The COVID-19 pandemic has shown us the need for effective, practical and inexpensive biomarkers.
- In univariate analysis, age, CRP, platelet count, PNI and albumin were found to be significant in showing the risk of mortality and ICU admission.
- Age, platelet count and CRP level were found to be independent risk factors for mortality and ICU admission.
- While creating the CAPA score, individual optimal cutoffs were determined for CRP level, age, platelet count and albumin level.
- Receiver operating characteristic analyses showed that CAPA score has highest area under the curve values.
- CAPA score is an effective scoring system in predicting mortality and ICU admission in COVID-19.
